# The Journal-Record the Official Organ of the Fulton County Medical Society

**Published:** 1906-03

**Authors:** 


### THE JOURNAL-RECORD THE OFFICIAL ORGAN OF THE FULTON COUNTY MEDICAL SOCIETY.

THE ATLANTA JOURNAL-RECORD OF MEDICINE has been constituted the official organ of the Fulton County Medical Society. The papers read before the Society, together with the discussions, will regularly appear in THE JOURNAL-RECORD. The program arranged for the ensuing year will indicate the amount of interesting material which is offered the readers of this Journal. The wide field covered by the papers will be almost equivalent to a postgraduate course, and we congratulate our readers upon their good fortune in being the recipients of the good things that have been provided for them.

This arrangement does not however limit the scope of the Journal to the narrow field of an organ of a medical society. It is merely a feature and a desirable addition to its contents, and does not place any limitations upon it.

---

THE standing committees of the Atlanta Board of Health have been announced by Dr. C. F. Benson, the president, as follows: Statistics—Brawner, chairman; Strickler and Vaughan. Finance—Robert, chairman; Johnson and Brawner. Plumbing and Drainage—Vaughan, chairman; Johnson and Mangum. Contagious and Infectious Diseases—Wolff, chairman; Strickler and Vaughan. Ordinances—Brandon, chairman; Roberts and Wolff. Purchasing Supplies—Johnson, chairman; Brandon and Mangum. Milk and Dairies—Mangum, chairman; Wolff and . Vaughan. Gathering and Destruction of Garbage—Strickler, chairman; Brandon and Mangum. Claims and Litigation—Vaughan, chairman; Brandon and Brawner.

---

DR. DE SASSURE FORD, of Augusta, Ga., died February 5th, at his home, after an illness of ten days, aged 71. Dr. Ford was a prominent citizen and an able physician. His position as Dean of the Medical College of the University of Georgia will be difficult to fill by such a competent man. His career was a long and honorable one and he was well known throughout the South as a surgeon of unusual skill.

---

BY an oversight, a selection, "The Physician as a Business Man," in our February issue was not credited to the *American Medical Journalist.* We take this opportunity of correcting the error.

